# Does repetitive transcranial magnetic stimulation have a beneficial effect on improving unilateral spatial neglect caused by stroke? A meta-analysis

**DOI:** 10.1007/s00415-024-12612-w

**Published:** 2024-08-28

**Authors:** Ruixuan Lin, Jack Jiaqi Zhang, Lingling Zhong, Sofina S. Y. Chan, Patrick W. H. Kwong, Lukas Lorentz, Usman Jawed Shaikh, Tommy L. H. Lam, David M. A. Mehler, Kenneth N. K. Fong

**Affiliations:** 1 Department of Rehabilitation Sciences, The Hong Kong Polytechnic University, Hung Hom, Hong Kong SAR China; 2 Division of Clinical Cognitive Sciences, Department of Neurology, University Hospital RWTH Aachen, Aachen, Germany; 3 University Research Facility in Behavioral and Systems Neuroscience, The Hong Kong Polytechnic University, Hung Hom, Hong Kong SAR China; 4 Department of Psychiatry, Psychotherapy and Psychosomatics, University Hospital RWTH Aachen, Aachen, Germany; 5 Institute for Translational Psychiatry, University of Münster, Münster, Germany

**Keywords:** Stroke, Unilateral spatial neglect, Repetitive transcranial magnetic stimulation, Meta-analysis, Rehabilitation

## Abstract

This review aimed to assess the effect of repetitive transcranial magnetic stimulation (rTMS) in improving post-stroke unilateral spatial neglect (USN) using a meta-analysis. Further, we aimed to identify any association between rTMS parameters, patient demographics, and treatment effect sizes using subgroup analyses and meta-regression. A literature search was conducted through four databases from inception to March 6, 2024, to retrieve all relevant controlled trials investigating the effects of rTMS on symptoms of USN in post-stroke patients. Overall, rTMS significantly improved post-stroke USN, as measured by the line bisection test (Hedges’ *g* = – 1.301, *p* < 0.0001), the cancelation test (Hedge’s *g* = – 1.512, *p* < 0.0001), and the Catherine Bergego Scale (Hedges’g = – 0.770, *p* < 0.0001), compared to sham stimulation. Subgroup analysis found that generally larger effect sizes following excitatory rTMS across several outcome measures, indicating that excitatory rTMS on the ipsilesional hemisphere may be more effective than inhibitory rTMS on the contralesional hemisphere in ameliorating neglect symptoms. Meta-regression analysis of the line bisection test showed a significant difference in the chronicity of stroke patients, suggesting that rTMS may be more effective for USN in patients at the acute stage (within 3 months since stroke) than in those at the post-acute stage (*p* = 0.035). In conclusion, rTMS appears to be effective in promoting recovery from post-stroke USN. Excitatory protocols and early intervention may enhance recovery outcomes for neglect behaviors in post-stroke survivors.

**Supplementary Information:**

The online version contains supplementary material available at 10.1007/s00415-024-12612-w.

## Introduction

Unilateral spatial neglect (USN) is a neuropsychological disorder manifested as an attention deficit to visual, auditory, or proprioceptive stimuli from the contralesional hemifield and is often observed in post-stroke patients [1], especially in those with right hemispheric lesions [11]. USN is also a strong predictor of neurological recovery after stroke, as recovery of cognitive and motor functions in post-stroke patients with USN is worse than in those without USN [9, 13]. USN is caused by disruption of the cortical attention network owing to brain injury, which consists of the dorsal attention network (DAN) and ventral attention network (VAN) [12]. The DAN includes the posterior parietal cortex (PPC) and frontal eye fields, which guide visual–spatial and visual-driven attentions. The VAN includes the right inferior frontal gyrus and right temporoparietal junction, which reorient attention to stimuli-driven covert visual-spatial attention. Damage to either area was associated with the occurrence of USN [10]. Researchers have proposed a model of spatial attention disturbance in patients with neglect, suggesting that they have an attentional bias toward the side of the space opposite the brain lesion. This bias is caused by an imbalance between the two attention-directing processes controlled by the right and left hemispheres. In a healthy brain, the competition between both hemispheres is mediated by inhibitory connections across the midline of the brain [5]. However, according to the imbalance hypotheses, disruption of long-range projections due to a brain lesion results in an imbalance that leads to a shift in attention and gaze toward the side of the brain lesion [29].

The mainstream rehabilitative intervention methods for post-stroke USN include prism adaptation (PA), visual scanning, optokinetic stimulation, and mirror therapy [45]. All these rehabilitative interventions activate the VAN via a bottom–up approach. Contrastingly, non-invasive brain stimulation promotes USN recovery by adjusting the balance of both brain hemispheres via top–down modulation [26]. Transcranial magnetic stimulation (TMS), the most commonly used non-invasive brain stimulation technique in post-stroke rehabilitation, uses the principle of electromagnetic induction to generate an electric current that acts on the neurons of the cerebral cortex, thereby affecting the metabolism and neuroelectric activity of the brain [31]. Repetitive transcranial magnetic stimulation (rTMS) has multifaceted effects on the nervous system. It influences the release of neurotransmitters and neurotrophic factors [36] and alters the functional connections between different brain regions [24]. Typically, high-frequency rTMS (HF-rTMS) and intermittent theta-burst stimulation (iTBS) induce an increase in excitability of the stimulated cortex, whereas low-frequency rTMS (LF-rTMS) and continuous theta-burst stimulation (cTBS) inhibit excitability in the stimulated cortex [30]. In clinical trials, rTMS is frequently used to treat USN by regulating the interhemispheric balance of attention system located in the bilateral parietal areas, through either inhibitory rTMS to the unaffected hemisphere or applying excitatory rTMS to the affected hemisphere, thus achieving a balance between the hemispheres to reduce the severity of USN. According to our previous review, rTMS yielded the largest effect size among interventions tailored to unilateral neglect [45].

Previous systematic reviews and meta-analyses have reported the positive effect of rTMS in improving USN in post-stroke patients [22, 26, 35, 44, 48]. However, the possible efficacy-related modulators on the treatment effects were not yet well explored in the previous reviews [22, 26, 35, 44, 48], which can be investigated by conducting meta-regression and subgroup analyses based on the meta-analytic results. Here, our review aimed to assess the effect of different rTMS protocols in improving post-stroke USN using a meta-analysis. Further, we aimed to identify any association between rTMS parameters, patient demographics, and treatment effect sizes using subgroup analyses and meta-regression.

## Methods

### Data sources and search strategy

The protocol of this review was registered in PROSPERO (CRD42024553592). Randomized controlled trials (RCTs) or nonrandomized controlled trials on the impact of rTMS in patients with USN after stroke were retrieved from PubMed, EMBASE, MEDLINE, and Web of Science databases from inception until March 6, 2024. The search strategy was developed as follows: (transcranial magnetic stimulation OR theta-burst stimulation) AND (unilateral neglect OR unilateral spatial neglect OR visuospatial neglect OR visuomotor neglect OR behavioral inattention OR hemispatial neglect) AND (stroke OR cerebrovascular accident OR cerebral infarction OR cerebral hemorrhage).

### Inclusion criteria

The inclusion criteria were developed in accordance with the PICOS framework: *Population (P):* adult patients (> 18 years old) with post-stroke USN. Intervention (I): using any rTMS protocols (HF-rTMS, LF-rTMS, cTBS, and iTBS). *Comparison (C):* sham stimulation or no rTMS. *Outcomes (O):* Studies must provide outcomes that reflect the severity of USN or the severity of USN affecting activities of daily living. The line bisection test (LBT) and star cancelation test (SCT) are the standard paper–pencil tests most used to assess the severity of USN. The Catherine Bergego Scale (CBS) is a standardized behavioral test of USN. These three tests were selected as primary outcomes of the current meta-analysis. When the LBT was not available, a subtest (bisection test) of the behavioral inattention test (BIT) was used in the meta-analysis. If the SCT was not available, the cancelation subtest (CT) of the BIT and the Albert test (AT), which are similar in nature to the SCT, were used for analysis. *Study design:* RCT or nonrandomized controlled trials.

### Exclusion criteria

This meta-analysis included only articles written in English language. Articles that did not use a scale measuring the degree of USN as an outcome, studies without any parallel control group (self-control, cohort studies, case–control studies, cross-sectional studies, animal experiments, expert consensus, conference abstracts, meta-analyses, or reviews), studies that did not use any form of therapeutic TMS protocols, and duplicate publications were excluded from consideration.

### Data extraction

Endnote version 21 was used to manage the citations. The extracted data included the first author, publication year, number of participants, mean age, sex, nature of lesion, stroke duration, treatment protocol, combined intervention, Class of studies, outcome measures, pre- and post-treatment means, mean change score, and standard deviations for outcome measures.

All included studies were classified into four classes based on the criteria used in the clinical guideline by Lefaucheur et al. [34]. If the mean and SD were not provided in the study, but the median and range of the score were available, this formulation was used: mean = (*a* + 2*m* + *b*)/4 (where a is the smallest value, b is the largest value, and m is the median); the standard deviation was calculated using the interquartile range/4 [23].

### Quality and risk-of-*bias* assessment

The PEDro scale was used to evaluate the reporting quality of the methodology [19]. The scale consists of 11 items, with the first item assessing the external validity of a study and the remaining items assessing internal validity. The PEDro Scale was scored from 0 to 10, with higher scores indicating higher research quality. A total score falling within the range of zero to three indicates low methodological quality, while a score of four-to-five accounts for moderate quality. When the score is between six and eight the quality is considered good. A score of nine or ten suggests excellent quality [15]. Two authors (RL and LZ) independently rated the PEDro scores for each study, and any disagreements were resolved through discussion with a senior author (JZ).

### Statistical analysis

Comprehensive meta-analysis (CMA) software (version 3.0) was used for the meta-analysis. The authors were contacted by email when the required data were missing. In case no response could be received from the authors, a data digitizer was used to extract the data obtained in the form of graphs. The data used in this analysis were continuous variables. The change score (postminus pre) and its standard deviation were used to compute the pooled effect size in the form of Hedge’s *g* [39]. The 95% confidence interval (CI) was calculated, and the significance level was set to α = 0.05. Effect sizes measured by Hedge’s *g* values of 0.15, 0.40, and 0.75 are interpreted as indicating small, medium, and large effects, respectively [3]. The *q*-statistics and *I*^2^ indices were used to evaluate the heterogeneity of each effect size. The random-effects model was used in all meta-analyses because of the significant clinical and statistical heterogeneity among the studies [2]. Due to the lack of follow-up data and different follow-up times in many of the included articles, we only focused on the effect of rTMS post-intervention in this meta-analysis. Subgroup analysis was performed according to the different rTMS protocols, Class of studies, and the time since stroke onset. We used 3 months as the cut-off, because the spontaneous biological recovery of USN is dominant within the first 3 months after stroke [33, 37], and this subgroup analysis was carried out to explore the possible differential effects between TMS applied on top of the spontaneous biological recovery trend post-stroke (within 3 months) and TMS applied when spontaneous biological recovery is largely diminished (over 3 months). Meta-regression was performed to investigate the association between various predictors and effect sizes (Hedges’ g). Predictors included in the meta-regression were mean age, sex (expressed as the percentage of female patients), the percentage of ischemic stroke patients, the baseline severity (baseline test score), and the rTMS parameters (the total number of applied pulses, the number of sessions, and the applied pulses each session). Sensitivity analysis was performed using the leave-one-out method to test the robustness of significant findings. Publication bias was investigated using funnel plots and the Egger’s regression test, with a significant level of *p* = 0.1.

## Results

### Study selection

**Fig. 1:**
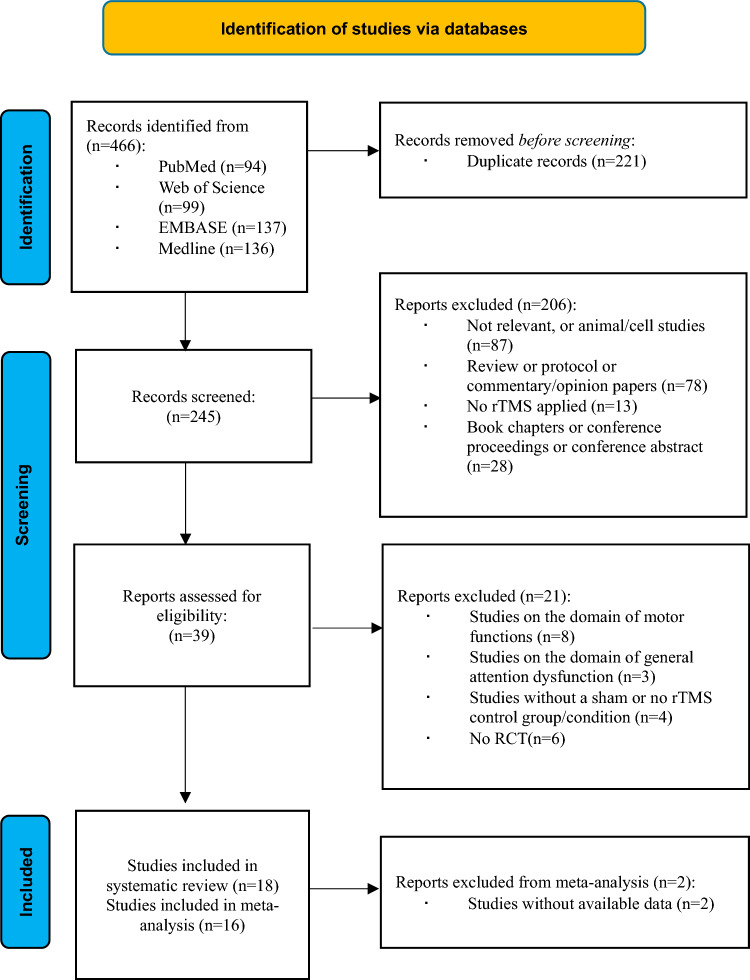
Flowchart of literature search

The search strategy identified 466 records in the database. After screening the records and according to the inclusion and exclusion criteria, 18 articles that met the inclusion criteria were included in the systematic review [4, 6–8, 16, 17, 21, 25, 27, 28, 32, 40–42, 46, 47, 49]. Two studies were excluded from the meta-analysis, because the data were not available; therefore, 16 studies were included in the meta-analysis [4, 6–8, 17, 25, 27, 28, 32, 40–42, 46, 47, 49]. The characteristics of the included studies are summarized in Table 1. The flowchart of the study selection is shown in Fig. 1.

**Table 1:** Characteristics of the included studies

		Population					rTMS protocol							Outcomes	Timeline
Study	Class of study	Group size	Chronicity	Age	Gender (female/male)	Nature of lesion	Frequency	Intensity	Sessions/duration	Number of pulses	Stimulation target	Control	Combined intervention		
Song et al. (2009) [40]	III	rTMS (*n* = 7) Sham (*n* = 7)	Acute	EG: 56.14 ± 8.99 CG: 64.43 ± 12.57	EG: 5/2 CG: 1/6	12 ischemic and 2 hemorrhagic stroke	0.5 Hz	90% MEP	20 sessions/2 times a day, 15 min each time	450	P3	Sham coil	Conventional rehabilitation	LCT; LBT	2 weeks before TMS; Baseline; Post; 2-week Post
Koch et al. (2012) [32]	II	cTBS (*n* = 10) Sham (*n* = 10)	Subacute	EG: 61.44 ± 13.02 CG: 71.89 ± 4.86	EG: 4/5 CG: 4/5	Ischemic stroke	cTBS: 50 Hz	80% AMT	10 sessions/2 times a day, 20 s each time	600	left PPC	90°flipped coil	Conventional rehabilitation	BIT-B; BIT-C	Baseline; Post; 2-week post
Cazzoli et al. (2012) [6]	II	cTBS then sham (*n* = 8) Sham then cTBS (*n* = 8) Sham (*n* = 8)	Subacute	EG1: 54.6 ± 11.8 EG2:60.7 ± 12.2 CG: 58.7 ± 12.7 All: 58.0	EG1: 6/14 EG2: 8/12 CG: 3/17 All: 7/17	41 ischemic and 19 hemorrhagic stroke	cTBS: 30 Hz	100% RMT	8 sessions/4 times a day, 44 s each time	801	P3	Sham coil	Conventional rehabilitation	CBS; RSCT; two part picture test;	Baseline; 1-week; 2-week; 3-week
Kim et al. (2013) [27]	II	EG1 = low-frequency rTMS (*n* = 6) EG2 = high-frequency rTMS (*n* = 6) CG = Sham rTMS (*n* = 6)	Acute	EG1: 68.6 ± 14.4 EG2: 64.1 ± 10.3 CG: 68.3 ± 6.5	EG1: 4/5 EG2: 5/4 CG: 3/6	23 ischemic and 4 hemorrhagic stroke	EG1: 1 Hz; EG2: 10 Hz	80% MT	10sessions/one session/ day, 20 min each session	EG1:1000 EG2:1200	EG1: P3; EG2: P4	90°flipped coil	Conventional rehabilitation	MVPT; CBS; LBT; SCT; K-MBI	Baseline; Post
Zhang et al. (2013) [49]	II	rTMS (*n* = 15) Sham (*n* = 15)	Acute	EG: 60 ± 10 CG: 57 ± 11	EG: 5/10 CG: 5/10	18 Ischemic stroke and 12 hemorrhagic stroke	0.5 Hz	90% RMT	20 sessions/2 times a day, 15 min/time	450	P3	Sham coil	Behavioral therapy	LBT; MBI; CD; AT	Baseline; Post
Cha et al. (2015) [8]	II	rTMS + CRT (*n* = 10) Sham + CRT (*n* = 6)	Acute	EG: 59.8 ± 9.9 CG: 56.7 ± 8.2	EG: 5/5 CG: 6/4	7 ischemic and 13 hemorrhagic stroke	1 Hz	90%MT	20 sessions/one session/day, 10 min/session	600	P3 and P4	Sham rTMS + sounds	Conventional rehabilitation	MVPT; LBT; AT; SCT	Baseline Post
Fu et al. (2015) [17]	II	cTBS + CRT (*n* = 10) sham cTBS + CRT (*n* = 10)	Acute to subacute	EG: 55.1 ± 13.95 CG: 59.5 ± 12.67	EG: 2/8 CG: 2/3	9 Ischemic stroke and 11 hemorrhagic stroke	cTBS: 30 Hz	80%RMT	56 sessions/four trains/day	600	P5	coil perpendicular to the patient’s scalp	Conventional rehabilitation	SCT; LBT	Baseline; 2-week; 6-week
Hopfner et al. (2015) [21]	III	SPT + cTBS (*n* = 6) SPT + sham cTBS (*n* = 6) Sham cTBS (*n* = 3) cTBS (*n* = 3)	Acute	EG: 62.0 ± 10.6 CG: 65.4 ± 15.2	EG: 7/5 CG: 2/4	13 ischemic stroke and 5 hemorrhagic stroke	cTBS: 30 Hz	100% RMT	2 sessions	801	P3	placebo coil	SPT	BCT; x-position of leftmost canceled target; Number of canceled targets; COC	Baseline; after SPT; after TMS
Yang et al. (2015) [48]	I	EG1 = 1 Hz rTMS (*n* = 9) EG2 = 10 Hz rTMS (*n* = 10) EG3 = cTBS (*n* = 9) CG = Sham (*n* = 10)	subacute	EG1: 46.72 ± 13.11 EG2: 48.01 ± 12.25 EG3: 49.45 ± 10.78 CG: 47.70 ± 11.81	EG1: 3/6 EG2: 6/4 EG3: 4/5 CG: 7/3	24 Ischemic stroke and 14 hemorrhagic stroke	EG1: 1 Hz EG2: 10 Hz EG3: cTBS: 30 Hz	80%RMT	20 sessions/2 times a day, 15 min/session	EG1 = 656 EG2 = 1000 EG3 = 801	P3	Back of the coil facing toward the stimulation point of the patient’s head	Conventional rehabilitation	SCT; LBT;	Two weeks before; Baseline; Post; One month post
Cao et al. (2016) [4]	III	iTBS (*n* = 7) Sham (*n* = 6)	Acute	EG: 55 ± 12 CG: 62 ± 10	EG: 1/6 CG: 1/5	Unclear	iTBS: 50 Hz	80% RMT	20 sessions/ Twice a day, 20 min/session	300	Left DLPFC, the F5 label of the left hemisphere	Sham coil + 40%RMT	Conventional rehabilitation	LBT; SCT	Baseline Post
Cha et al. (2016) [7]	II	rTMS + CRT (*n* = 15) Sham + CRT (*n* = 15)	Acute	EG: 64.07 ± 12.1 CG: 63.33 ± 12.16	EG: 8/7 CG: 6/9	18 ischemic and 12 hemorrhagic stroke	1 Hz	90% MT	20 sessions/ one session/day, 10 min/session	1200	P3	Sham rTMS + sounds	Conventional rehabilitation	LBT; AT;	Baseline Post
Fu et al. (2017) [16]	III	cTBS (*n* = 6) Sham (*n* = 6)	Acute	EG: 60.17 ± 14.0 CG: 62.00 ± 9.785	All: 3/9	hemorrhagic stroke	cTBS:30 Hz	80%RMT	40 sessions/ Four sessions/day	600	P3	coil placed perpendicular to the scalp + 40%RMT	Conventional rehabilitation	SCT; LBT	Baseline; Post
Yang et al. (2017) [46]	I	EG1 = rTMS combined with sensory cueing (*n* = 20) EG2 = rTMS group (*n* = 20) CG = Conventional (*n* = 20)	Acute	EG1: 54.6 ± 11.8 EG2: 60.7 ± 12.2 CG:58.7 ± 12.7	EG1: 6/14 EG2: 8/12 CG: 3/17	41 ischemic and 19 hemorrhagic stroke	1 Hz	80%RMT	10 sessions/one session/day	900	P5	No	Conventional rehabilitation	MBI; BIT-Cancelation tasks; BIT-C; CBS; BIT-Drawing tasks	Baseline; Post; 1-month post
Kim et al. (2018) [28]	II	CG: robot therapy (*n* = 10) EG1: rTMS (*n* = 10) EG2: robot therapy combined with rTMS (*n* = 10)	Acute	EG1: 70.3 ± 9.6 CG: 66.6 ± 12.2 EG2: 62.5 ± 16.5	EG1: 5/5 CG: 5/5 EG2: 5/5	16 ischemic and 14 hemorrhagic stroke	0.9 Hz	95% MT	10 sessions/one session/day, 20 min/session	900	position P5 localized over the left posterior parietal cortex	No	Robot therapy	MVPT-3; LBT; SCT; K-MBI; CBS; AT	Baseline; Post
Nyffeler et al. (2019) [37]	II	EG1 = 8cTBS (*n* = 10) EG2 = 16cTBS (*n* = 10) CG = Sham (*n* = 10)	Subacute	EG1: 67.8 ± 10.13 EG2: 74.3 ± 10.23 CG: 70.60 ± 11.44	EG1: 5/5 EG2: 4/6 CG: 7/3	Not reported	cTBS:30 Hz	100%RMT	8/16 sessions/ 44 s/time 4 times per day for 2/4 days	801	P3	Sham coil	Conventional rehabilitation	CBS; FIM;	Baseline; Post 10 sessions Post 20 sessions; 3-month post
Vatanparast et al. (2019) [42]	II	cTBS (*n* = 7) Sham (*n* = 7)	Acute to subacute: 7 Chronic: 7	EG: 67.5 ± 8.4 CG: 65.5 ± 10.2	EG: 4/3 CG: 4/3	6 ischemic and 8 hemorrhagic stroke	cTBS:30 Hz	80% RMT	10 sessions/one session/day	801	P3	90°flipped coil	prism adaptation (PA)	SCT; LBT; FCT; CD	Baseline; Post; 3 months post
Iwan ´ski et al. (2020) [25]	II	rTMS (*n* = 14) sham rTMS (*n* = 14)	Acute	EG: 65 ± 7.5 CG: 64.6 ± 7.7	EG: 3/11 CG: 3/11	26 ischemic and 2 hemorrhagic stroke	1 Hz	90% RMT	15session/ one session/day, 30-min/session	1800	left angular gyrus	Sham coil and sounds	Visuos spatial training and conventional rehabilitation	BIT-c; BIT-b; VSS	Baseline; Post; 3 months post
Vatanparast et al. (2023) [41]	III	cTBS (*n* = 7) Sham (*n* = 7)	Acute to subacute: 7 Chronic: 7	CG: 65.42 ± 9.98 EG: 67.57 ± 8.44	Not reported	6 ischemic and 8 hemorrhagic stroke	cTBS:30 Hz	80%RMT	10 sessions/one session/day	801	P3	90°flipped coil	prism adaptation	LBT; FCT; CT	Baseline; Post

*AT* Albert Test, *BIT-B* Behavioral Inattention Test Behavioral subtest, *BCT* Bird Cancelation Task, *BIT-C* Behavioral Inattention Test Conventional subtest, *CBS* Catherine Bergego Scale, *cTBS* continue theta-burst stimulation, *CD* Clock Drawing, *COC* Center of Cancelation, *EG* experimental group, *FIM* Function Independent Measure, *FCT* Figure Copying Test, *iTBS* Intermittent theta-burst stimulation, *K-MB* Korean version of Modified Barthel, *LBT* line bisection test, *MBI* Modified Barthel Index, *MT* motor threshold, *MRT* Munich reading texts, *MVPT* Meaningful Visual Perception Test, *PPC* posterior parietal cortex, *RMT* resting motor threshold, *rTMS* repetitive transcranial magnetic stimulation, *RSCT* random shape cancelation test, *SCT* star cancelation test, *SPT* Smooth pursuit eye movement training, *VSS* Visuospatial Scale

### Methodological quality assessment

The quality of the included articles was rated using the PEDro scale (Table S1). The mean score of the 18 articles was 8.61, ranging from 5 to 10. This indicated that the included articles were of moderate-to-high quality.

### rTMS protocols

Most studies (*n* = 9) used LF-rTMS, four of which used the 1-Hz frequency for the P3 based on the EEG 10–20 system [7, 8, 27, 47] and the other two also used the same frequency while applying it to the contralesional angular gyrus [25], or P5 [28, 46]. Other three studies also chose to use LF-rTMS, but they applied different frequencies: two studies used 0.5 Hz rTMS to the P3 [40, 49]. One study applied 0.9 Hz rTMS to the P5 [28]. High-frequency rTMS (10 Hz) was used in two studies, separately acting on the P3 [47] or P4 [27]. cTBS was used in nine studies, the stimulation target was set at the P3 in eight studies [6, 16, 21, 32, 41, 42, 47], and one study chose the P5 as the stimulation target [17]. Only one study [4] used the iTBS protocol for the left DLPFC, which was localized by the F5 channel in the EEG 10–20 system.

### Effect of rTMS on unilateral spatial neglect

#### Line bisection test

**Fig. 2:**
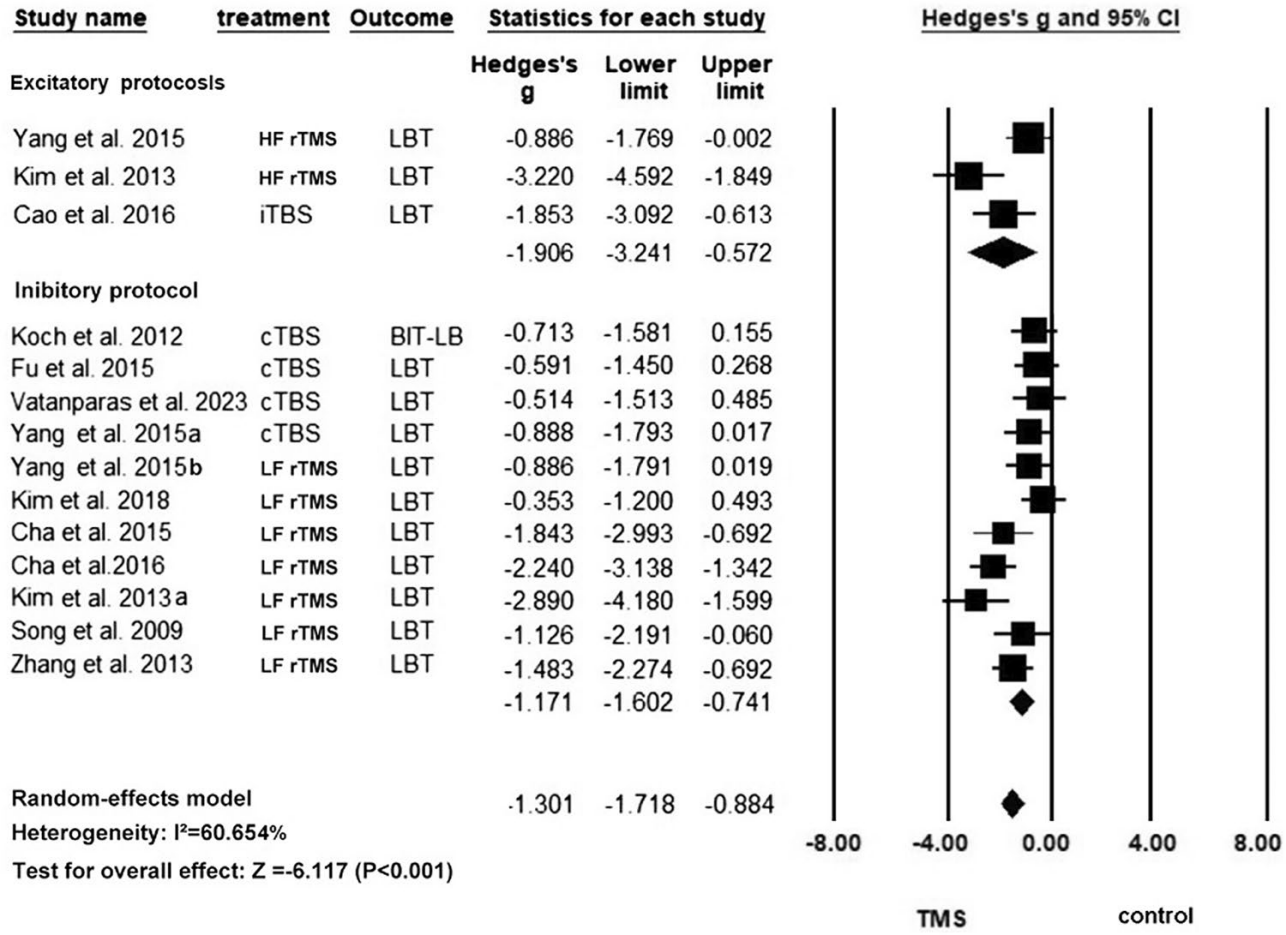
Meta-analysis for LBT

**Fig. 3:**
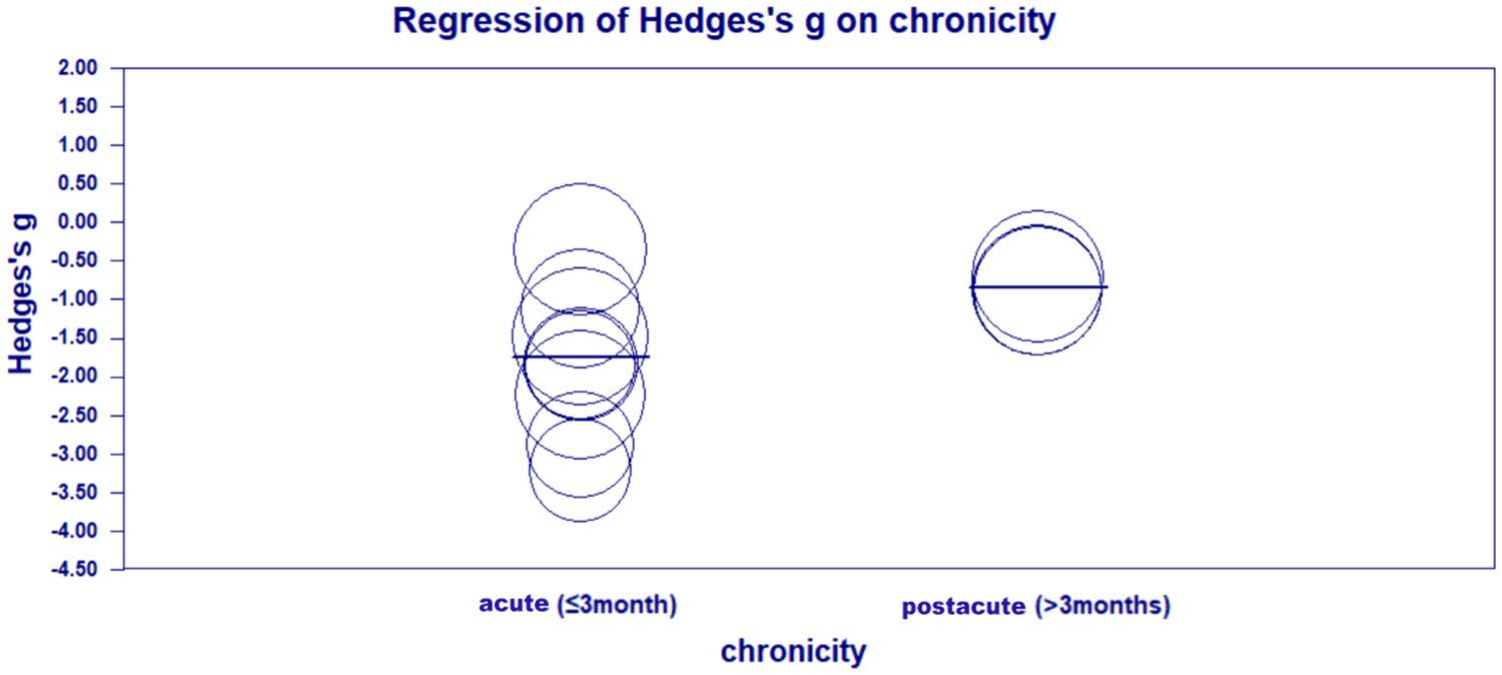
Between-group differences in the effect sizes of LBT in acute and postacute patients

Eleven studies with 14 units of analysis were included in this meta-analysis of LBT [4, 7, 8, 17, 27, 28, 32, 40] (Fig. 2). An overall improvement in the LBT score was found in the rTMS group compared with the control group (Hedges’ *g* = – 1.301, *p* < 0.0001, *I*^2^ = 60.65%), and the overall significance was robust to the leave-one-out sensitivity analysis (Hedge’s *g* from – 0.884 to – 1.718, which consistently indicated a large effect size in our sensitivity analysis). Subgroup analyses showed that the excitatory and inhibitory rTMS protocols both significantly improved USN. The pooled effect size for excitatory rTMS stroke acute was numerically larger than the inhibitory rTMS (excitatory: Hedge’s *g* = – 1.906, *p* = 0.005, *I*^2^ = 75.05%; inhibitory: Hedges’ *g* = – 1.171, *p* < 0.0001, *I*^2^ = 56.33%). There was sign of publication bias according to the significant results of Egger’s test (*p* = 0.010) (Figure S1). Univariate meta-regression analysis (Fig. 3) showed that the post-stroke period of patients (acute or post-acute) was a significant predictor of the effect size, and rTMS appeared to be more effective for patients in the acute phase (*p* = 0.035) than those in the post-acute phase. Other predictors were not significant in the meta-regression. The subgroup analysis based on the classes of studies shows no significant difference in the effect sizes between subgroups (*Q* = 1.07, *p* = 0.586). Table 2 summarizes the results of the univariate meta-regression.

**Table 2:** Results of meta-regression of moderators for the effect sizes of rTMS in LBT scores

Moderators	*N*	Univariate coefficient	*Z* value	*P* value
rTMS parameters				
Number of rTMS session	14	0.0158	0.85	0.3979
Total pulses	14	0.0000	0.58	0.5641
Number of pulses per session	14	– 0.0011	– 1.34	0.1809
Demographics				
Mean age (years)	13	– 0.0300	– 0.99	0.3207
Percentage of female patients	13	0.7788	0.40	0.6908
Clinical profiles				
Percentage of ischemic stroke patients	13	– 1.2823	– 1.03	0.3046
Mean baseline severity (LBT)	12	– 0.0033	– 0.35	0.7271
Chronicity (acute/post-acute)	12	0.9149	2.10	0.0354*

^*^p < 0.05; **p < 0.01; ***p < 0.001

#### Cancelation test

**Fig. 4:**
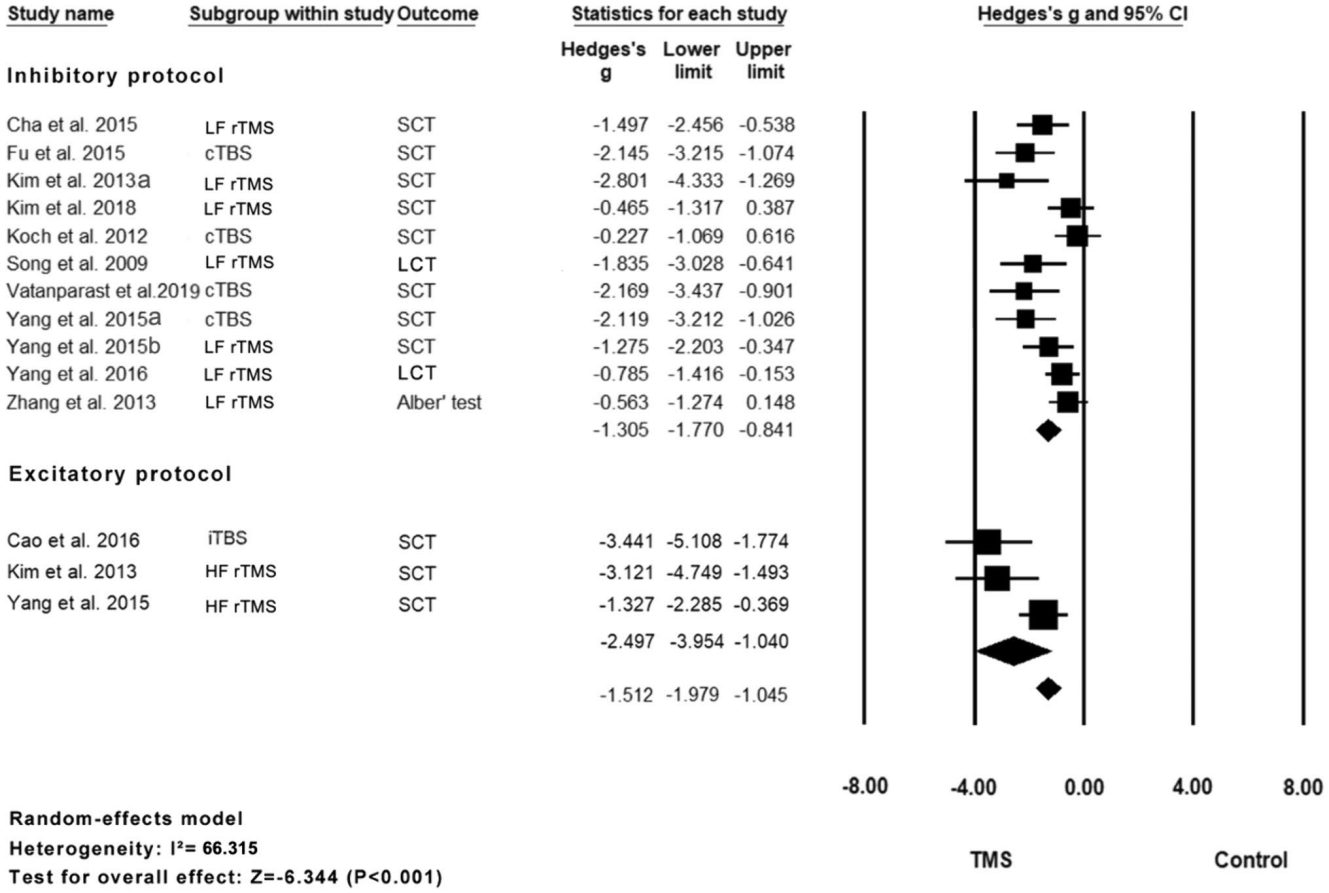
Meta-analysis for CT

Eleven studies with 14 units of analysis were included in the meta-analysis of the cancelation test scores [4, 8, 17, 27, 28, 32, 40, 42, 46, 47, 49] (Fig. 4). The results indicated that the rTMS group showed a significant improvement in scores compared with the control group (Hedge’s *g* = – 1.512, *p* < 0.0001, *I*^2^ = 66.32%). The overall effect was still robust in the leave-one-out sensitivity analysis (Hedge’s *g* from – 1.979 to – 1.405, which consistently indicated a large effect size in our sensitivity analysis). There was a sign of publication bias according to the significant results of Egger’s test (*p* = 0.020) (Fig. S2). The subgroup analysis showed that the excitatory rTMS group had a larger effect size than the inhibitory rTMS subgroup, and both were statistically significant (excitatory: Hedge’s *g* = – 2.497, *p* = 0.001, *I*^2^ = 69.22%; inhibitory: Hedges’ *g* = – 1.305, *p* < 0.0001, *I*^2^ = 61.68%). The subgroup analysis based on the classes of studies shows no significant difference in the effect sizes between subgroups (*Q* = 2.18, *p* = 0.336). Table 3 summarizes the results of the univariate meta-regression.

**Table 3:** Results of meta-regression of moderators for the effect sizes of rTMS in CT scores

Moderators	N	Univariate coefficient	Z value	P value
rTMS parameters				
Number of rTMS session	14	– 0.0185	– 0.91	0.3605
Total pulses	14	– 0.0000	– 0.96	0.3381
Number of pulses per session	14	– 0.0004	– 0.34	0.7368
Demographics				
Mean age (years)	13	0.0008	0.02	0.9836
Percentage of female patients	13	0.5546	0.27	0.7875
Clinical profiles				
Percentage of ischemic stroke patients	13	0.3732	0.29	0.7711
Mean baseline severity (CT)	12	– 0.0122	– 1.07	0.2863
Chronicity (acute/post-acute)	14	0.3375	0.67	0.5001

#### Catherine Bergego scale

**Fig. 5:**
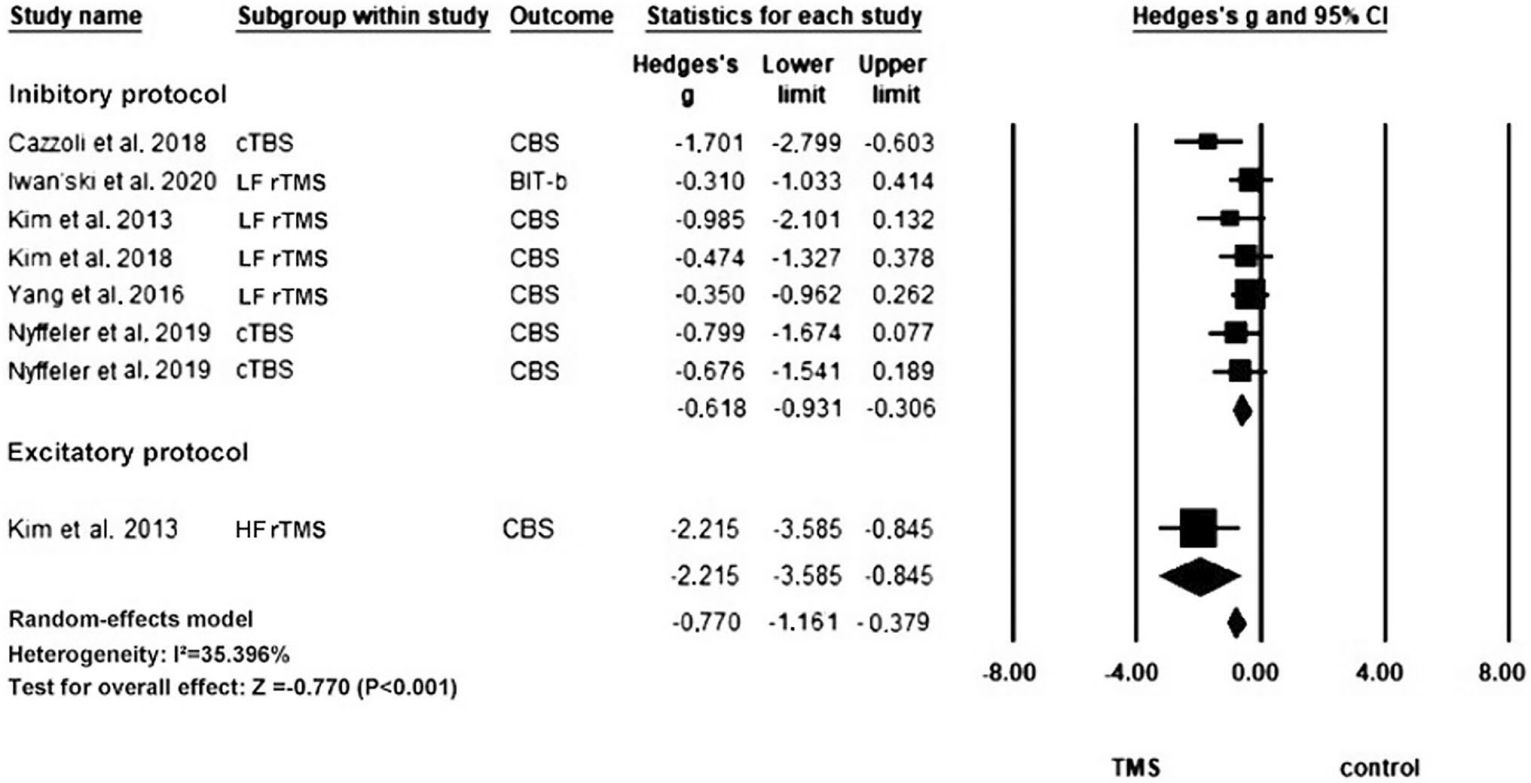
Meta-analysis for CBS

In total, six articles with eight units of analysis were included in this meta-analysis of CBS [6, 21, 25, 27, 28, 46] (Fig. 5). The rTMS group showed a significant improvement in the CBS score compared with the control group (Hedges’ *g* = – 0.770, *p* < 0.0001, *I*^2^ = 35.40%), and this overall significance remained robust in the leave-one-out sensitivity analysis (Hedge’s *g* from – 0.379 to – 1.161, which showed a medium-to-large effect size in our sensitivity analysis). There was a sign of publication bias according to the significant results of Egger’s test (*p* = 0.010) (Fig. S3). Subgroup analysis revealed that both excitatory and inhibitory rTMS protocols led to significant improvements in the behavioral test results of USN. However, the effect size for excitatory rTMS was numerically larger than that for inhibitory rTMS (Excitatory: Hedges’ *g* = – 2.215, *p* = 0.002, *I*^2^ = 0.00%; inhibitory: Hedges’ *g* = – 0.618, *p* < 0.0001, *I*^2^ = 0.00%). The subgroup analysis based on the classes of studies shows no significant difference in the effect sizes between subgroups (*Q* = 4.87, *p* = 0.431). Table 4 summarizes the results of the univariate meta-regression.

**Table 4:** Results of meta-regression of moderators for the effect sizes of rTMS in CBS scores

Moderators	*N*	Univariate coefficient	*Z* value	*P* value
rTMS parameters				
Number of rTMS session	8	0.0616	0.85	0.3935
Total pulses	8	0.0000	0.86	0.3888
Number of pulses per session	8	0.0004	0.59	0.5579
Demographics				
Mean age (years)	8	– 0.0131	– 0.49	0.6227
Percentage of female patients	8	– 0.8691	– 0.51	0.6108
Clinical profiles				
Percentage of ischemic stroke patients	6	– 0.2884	– 0.14	0.8896
Mean baseline severity (CBS)	5	0.0037	0.56	0.5724
Chronicity (acute/post-acute)	8	0.0003	0.00	0.9994

### Combined intervention

In total, 11 out of 18 studies included in this review utilized a combination of conventional rehabilitation and rTMS. However, other studies have opted for different approaches, such as smooth pursuit eye movement training [21], robot therapy for simultaneous visual scanning and limb activation [28], and sensory cueing using a wearable device [46]. Additionally, two studies employed PA [41, 42]. All these interventions demonstrated effectiveness when combined with rTMS to improve USN.

#### Level of recommendation

We summarized the level of recommendation of the efficacy of different rTMS protocols in treatment of post-stroke neglect, following the definition of Lefaucheur et al. [34]. The level of recommendation in the efficacy of LF-rTMS has now reached level A, with two Class I studies [46, 47], six Class II studies [7, 8, 25, 27, 28, 49], and one Class III study [40]. The level of recommendation in the efficacy of cTBS has now reached Level B, with one Class I study [47], five Class II studies [6, 17, 32, 42], and three Class III studies [16, 21, 41]. The level of recommendation in the efficacy of HF-rTMS has now reached level C, with one Class I study [47] and one Class II study [27]. No recommendation for iTBS can be made as only one experiment [4] was available. The two excitatory proposals (HF-rTMS and iTBS) have not received high recommendation levels due to the limited number of studies at this stage; however, the numerically larger effect size observed in meta-analyses to some extent can support that the excitatory protocols have great potential in the treatment of post-stroke USN.

## Discussion

Our study found that (1) rTMS was significantly effective in improving post-stroke USN compared with the control group. (2) Excitatory rTMS appears to be more effective than inhibitory rTMS, with a numerically larger effect size than that of inhibitory protocols; however, a few studies have utilized excitatory rTMS and the level of recommendation is thereby lower when compared to inhibitory protocols. (3) rTMS seems to effectively improve neglect behaviors during daily activities in post-stroke patients. (4) A significant difference was found between the chronicity of stroke patients and the effect size of rTMS in the LBT meta-regression, indicating that the timing of stroke may be a factor influencing the efficacy of rTMS, with patients in the acute phase (within the first 3 months) potentially benefiting more from the non-invasive brain stimulation therapy.

Recovery from neglect after stroke depends on neuroplasticity [18]. Spontaneous biological recovery, which is dominant with the first three months after stroke, significantly contributes to the spontaneous recovery from USN. Our analysis suggested that the timing of rTMS intervention may be a potential factor influencing the outcome of USN after stroke. Our analysis showed that rTMS delivered within the first three months post-stroke demonstrated a significantly stronger effect on facilitating the recovery from USN, compared with rTMS applied after the first three months. The results indicated that rTMS may have an add-on effect on spontaneous biological recovery from USN in post-stroke survivors. However, this significant difference was only observed in the meta-analysis of LBT, perhaps due to the differences among the neglect measures [20]. LBT necessitates the correct perception of the size of a single stimulus, while CT depends on a normal visual search within an array of various stimuli [14]. CBS is related to daily activities; therefore, it requires higher cognitive function. This finding may suggest that the add-on benefit from rTMS on spontaneous recovery from USN is more specific to basic attention to a single stimulus.

Functional connectivity between the bilateral attention systems is assumably disrupted in post-stroke patients with USN [38]. Inhibitory protocols applied to the contralesional attention system can suppress the interhemispheric inhibition from the contralesional to the ipsilesional attention system, therefore facilitating recovery from USN. However, its effect depends on intact interhemispheric connectivity [37]. The effect of inhibitory rTMS may be therefore limited in patients with an injured corpus callosum, although we were unable to perform a quantitative analysis based on the integrity of the corpus callosum, because this information was not usually reported in the previous trials. In contrast, excitatory rTMS can promote the activation of adjacent functional areas through the parieto-frontal attention network in the affected hemisphere, such as the temporoparietal junction and the inferior frontal gyrus [27]. Besides, excitatory rTMS on the same hemisphere may not only promote the recovery of visual-spatial attention, but also potentially facilitate the recovery of other non-spatial functions, such as alertness and novelty detection[43]. Therefore, the excitatory protocol might yield a more consistent treatment response in USN.

### Limitations

This study also has some limitations. First, the rTMS protocols used in the included studies varied in treatment duration, target points, frequency, etc., which may have led to high heterogeneity. Second, in this meta-analysis, different scaling methods used for the same test were not considered for grouping. For example, LBT can be performed using both five- and one-line methods. Additionally, due to the unavailability of information on lesion location and specific types of USN for all participants, further analyses could not be conducted. Third, there is a risk of publication bias according to our analysis. In addition, the small sample size of the included studies, as well as the exploratory nature and lack of follow-up data, may downgrade the level of evidence in this field. Future pre-registered studies using large sample sizes are needed to verify the current findings. Finally, our meta-analyses were limited to published aggregate data. Mega-analyses using individual data will allow further investigation but require data sharing, for instance through large consortia such as ENIGMA.

## Conclusion

rTMS has shown promise as a potential treatment for facilitating recovery from post-stroke USN. Furthermore, generally larger effect sizes following excitatory rTMS across several outcome measures suggest that excitatory rTMS on the ipsilesional hemisphere may be more effective than inhibitory rTMS on the contralesional hemisphere in ameliorating neglect symptoms. Early delivery of rTMS treatment may yield a more favorable recovery outcome in neglect behaviors in post-stroke patients by accelerating the spontaneous recovery from USN within the first 3 months.

## Supplementary Information

Below is the link to the electronic supplementary material.

Supplementary file1 (DOCX 474 KB)

## Data Availability

Data supporting the findings of this study are available from the corresponding author on reasonable request.
