# Supplementary material for: Does repetitive transcranial magnetic stimulation have a beneficial effect on improving unilateral spatial neglect caused by stroke? A meta-analysis
Source: J Neurol. 2024 Aug 28;271(10):6494–507. doi: 10.1007/s00415-024-12612-w (PMC11446973; doi:10.1007/s00415-024-12612-w)

**Does repetitive transcranial magnetic stimulation have a beneficial effect on improving unilateral spatial neglect caused by stroke? A meta-analysis**

Ruixuan LIN*^1,^ MSc

Jack Jiaqi ZHANG^1*#^, PhD

Lingling ZHONG^1,^ MSc

Sofina SY Chan^1,^ MA

Patrick WH KWONG^1^, PhD

Lukas LORENTZ^2^, PhD

Usman Jawed SHAIKH^2^, MSc

Tommy LH Lam^3^, PhD

David MA MEHLER^4,5^, MD, PhD

Kenneth NK FONG^1^, PhD

1Department of Rehabilitation Sciences, The Hong Kong Polytechnic University, Hong Kong SAR, China.

^2^Division of Clinical Cognitive Sciences, Department of Neurology, RWTH Aachen University, Aachen, Germany

^3^University Research Facility in Behavioral and Systems Neuroscience, The Hong Kong Polytechnic University, Hong Kong SAR, China.

^4^Department of Psychiatry, Psychotherapy and Psychosomatics, University Hospital RWTH Aachen, Aachen, Germany

^5^Institute for Translational Psychiatry, University of Münster, Münster, Germany.

*Co-first authorship

**Figure S1: Flowchart of literature search.**

Records identified from (n=466):

- PubMed (n=94)
- Web of Science (n=99)
- EMBASE (n=137)
- Medline (n=136)

Records removed *before screening*:

- Duplicate records (n=221)

Records screened:

(n=245)

Reports assessed for eligibility:

(n=39)

Reports excluded (n=206):

- Not relevant, or animal/cell studies (n=87)
- Review or protocol or commentary/opinion papers (n=78)
- No rTMS applied (n=13)
- Book chapters or conference proceedings or conference abstract (n=28)

Studies included in systematic review (n=18)

Studies included in meta-analysis (n=16)

**Identification of studies via databases**

**Identification**

**Screening**

**Included**

Reports excluded (n=21):

- Studies on the domain of motor functions (n=8)
- Studies on the domain of general attention dysfunction (n=3)
- Studies without a sham or no rTMS control group/condition (n=4)
- No RCT(n=6)

Reports excluded from meta-analysis (n=2):

- Study without available data (n=2)

| **Study** | **Item1** | **Item2** | **Item3** | **Item4** | **Item5** | **Item6** | **Item7** | **Item8** | **Item9** | **Item10** | **Item11** | **Total score** |
| --- | --- | --- | --- | --- | --- | --- | --- | --- | --- | --- | --- | --- |
| Song et al., 2009 | 1 | 1 | 1 | 1 | 1 | 0 | 0 | 1 | 1 | 1 | 1 | 8 |
| Koch et al., 2012 | 1 | 1 | 1 | 1 | 1 | 1 | 1 | 1 | 1 | 1 | 1 | 10 |
| Kim et al., 2013 | 1 | 1 | 1 | 1 | 1 | 1 | 1 | 1 | 1 | 1 | 1 | 10 |
| Zhang et al., 2013 | 1 | 1 | 0 | 1 | 1 | 0 | 0 | 1 | 1 | 1 | 1 | 7 |
| Cha et al., 2015 | 1 | 1 | 1 | 1 | 1 | 1 | 1 | 1 | 1 | 1 | 1 | 10 |
| Fu et al., 2015 | 1 | 1 | 0 | 1 | 1 | 0 | 0 | 1 | 1 | 1 | 1 | 10 |
| Hopfner et al., 2015 | 1 | 0 | 0 | 1 | 0 | 0 | 0 | 1 | 1 | 1 | 1 | 5 |
| Yang et al., 2015 | 1 | 1 | 1 | 1 | 1 | 1 | 0 | 1 | 1 | 1 | 1 | 9 |
| Cao et al., 2016 | 1 | 1 | 1 | 1 | 1 | 0 | 0 | 1 | 1 | 1 | 1 | 8 |
| Cha et al., 2016 | 1 | 1 | 1 | 1 | 1 | 1 | 1 | 1 | 1 | 1 | 1 | 10 |
| Fu et al., 2017 | 1 | 1 | 1 | 1 | 1 | 1 | 1 | 1 | 1 | 1 | 1 | 7 |
| Yang et al., 2017 | 1 | 1 | 1 | 1 | 1 | 1 | 0 | 1 | 1 | 1 | 1 | 9 |
| Cazzoli et al., 2018 | 1 | 1 | 0 | 1 | 1 | 0 | 0 | 1 | 1 | 1 | 1 | 9 |
| Kim et al., 2018 | 1 | 1 | 1 | 1 | 1 | 1 | 1 | 1 | 1 | 1 | 1 | 10 |
| Nyffeler et al., 2019 | 1 | 1 | 1 | 1 | 1 | 0 | 0 | 1 | 1 | 1 | 1 | 8 |
| Vatanparast et al., 2019 | 1 | 1 | 1 | 1 | 1 | 0 | 0 | 1 | 1 | 1 | 1 | 8 |
| Iwan ́ski et al., 2020 | 1 | 1 | 1 | 1 | 1 | 0 | 1 | 1 | 1 | 1 | 1 | 9 |
| Vatanparast et al., 2023 | 1 | 1 | 1 | 1 | 1 | 0 | 0 | 1 | 1 | 1 | 1 | 8 |

**Table S1: Article quality assessment result**

**Figure S2: The funnel plot for the meta-analysis regarding outcome of LBT**


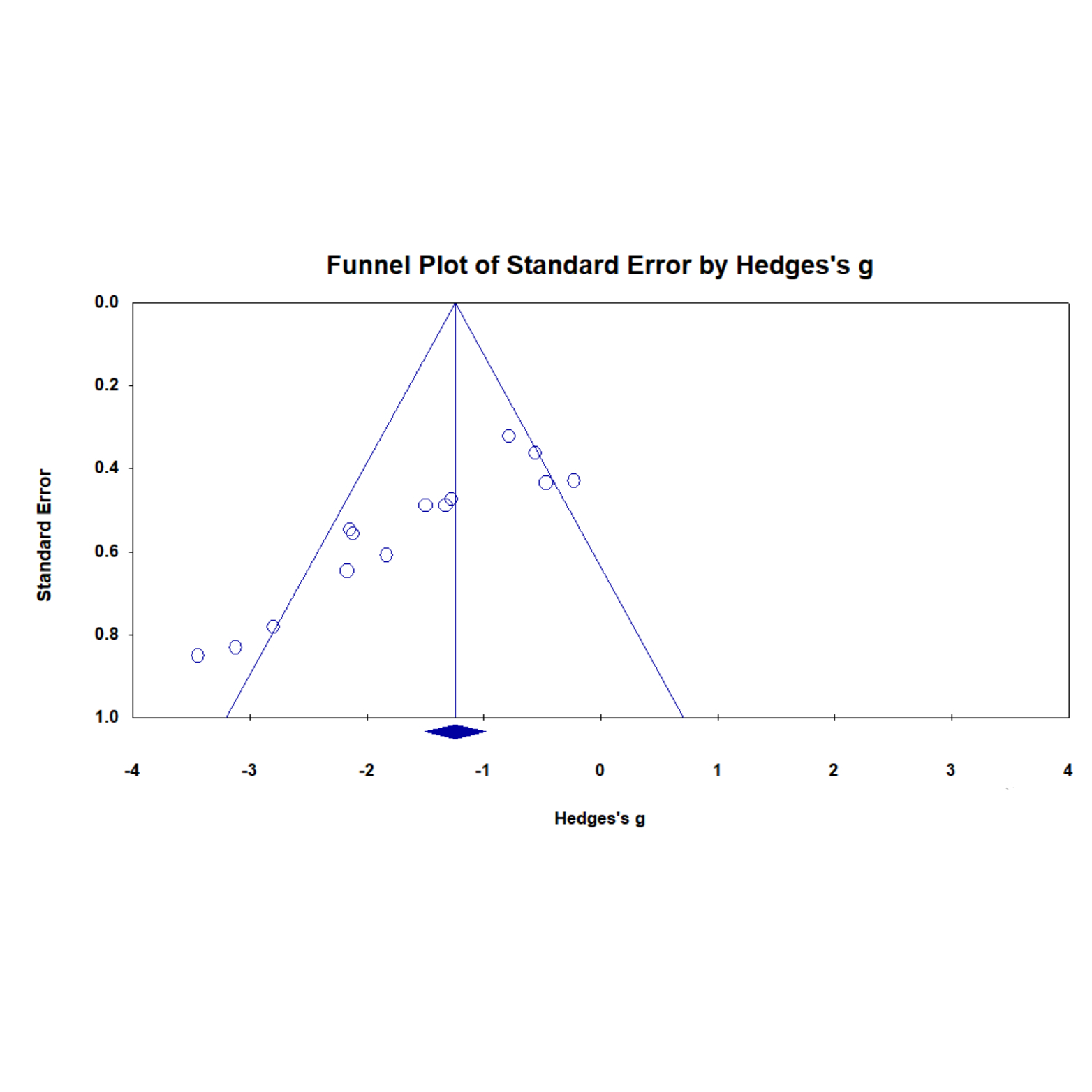


**Figure S3: The funnel plot for the meta-analysis regarding outcome of CT**


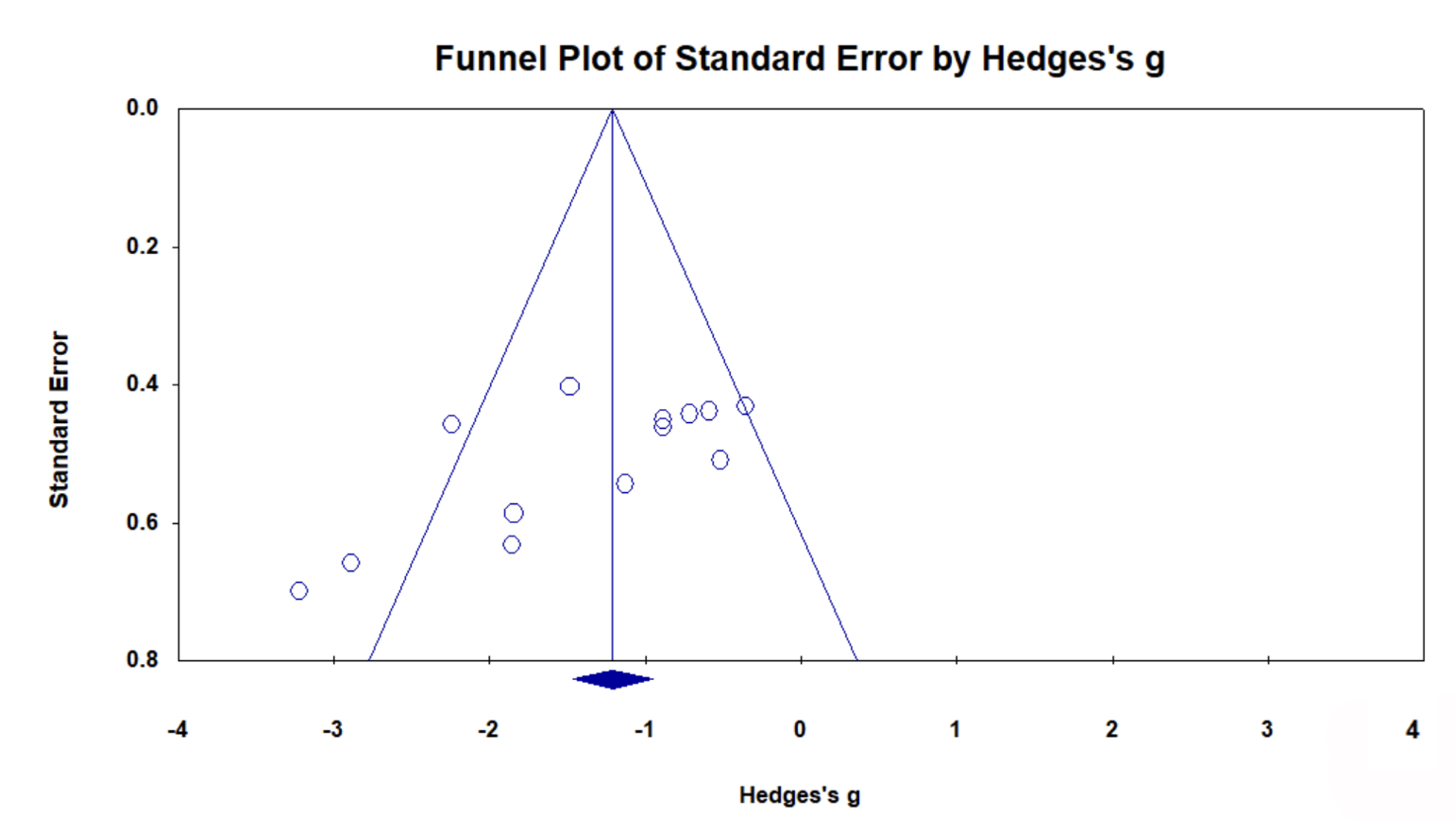


**Figure S4: The funnel plot for the meta-analysis regarding outcome of CBS**


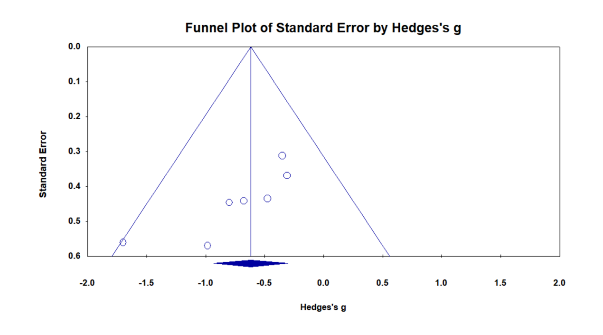

Supplement: Supplementary file 1 — Supplementary file1 (DOCX 474 KB) [file 415_2024_12612_MOESM1_ESM.docx]
